# Relationship Between the Cortisol-Estradiol Phase Difference and Affect in Women

**DOI:** 10.5334/jcr.154

**Published:** 2018-02-21

**Authors:** Karyn Geralyn Butler

**Affiliations:** 1 Grand Valley State University, US

**Keywords:** circadian, cortisol, estradiol, phase difference, affect

## Abstract

Affective disorders impact women’s health, with a lifetime prevalence of over twelve per cent. They have been correlated with reproductive cycle factors, under the regulation of hormonal circadian rhythms. In affective disorders, circadian rhythms may become desynchronized. The circadian rhythms of cortisol and estradiol may play a role in affective disorders. The purpose of this study was to explore the temporal relationship between the rhythms of cortisol and estradiol and its relationship to affect. It was hypothesized that a cortisol-estradiol phase difference (PD) exists that correlates with optimal affect. A small scale, comparative, correlational design was used to test the hypothesis. Twenty-three women were recruited from an urban university. Salivary samples were collected over a twenty-four-hour period and fitted to a cosinor model. Subjective measures of affect were collected. Relationships between the cortisol-estradiol PD and affect were evaluated using a second-degree polynomial equation. Results demonstrated a significant correlation in affect measures (*p* < 0.05). An optimal PD was identified for affect to be 3.6 hours. The phase relationship between cortisol and estradiol may play a role in the development of alterations in affective disorders.

## Background

Affective disorders impact the health of women world-wide, with a lifetime prevalence in women of over 12 per cent [1]. Affect is the emotional process experienced by individuals representing their psychological mood dispositions. Affect itself is the subjective and objective experience of emotions. Affect is closely associated with mood disorders including depression and anxiety. Depression can be characterized by low positive affect and high negative affect. Women have higher rates of depression than men, which is unrelated to response and recall biases but may be related to sex hormones, genes, or gendered social roles [2]. Affective disorders have been correlated with reproductive cycle factors, such as the use of oral contraceptives, the luteal phase of the menstrual cycle, postpartum, and menopause [3]. Research suggests that in affective disorders, circadian rhythms may become desynchronized [4,5].

Proper functioning of the human circadian system relies on synchronicity between the master clock, the suprachiasmic nuclei (SCN), and peripheral oscillators. It has been demonstrated in animal and in-vitro studies that peripheral clocks can maintain their rhythm independent of the SCN control [6]. This independence can alter the organization of the body’s circadian rhythms. This happens when peripheral oscillators become desynchronized from the SCN. Effects of desychrony among internal circadian rhythms may contribute to the development of adverse health states. Studies support a relationship between circadian rhythm desynchrony and diseases. Cancer [7], metabolic disorders [8], immune dysfunction, inflammatory and stress responses [9] have been correlated with desynchronized rhythms.

The purpose of this study was to investigate the relationship between the synchrony of estradiol and cortisol, two hormonal rhythms, and affect in premenstrual white women.

Under natural environmental conditions, circadian rhythms are maintained to a 24-hour period by strong and weak entrainers [10,11]. The most potent environmental entrainer is believed to be light. Animal model studies have revealed that peripheral circadian rhythms can be desynchronized by many mechanisms including the timing of feedings [12], activity and sleep [9], stress [13], body temperature and cortisol [14]. Areas where desynchrony has been shown to occur include tissue protein production in the hypothalamus [15], liver [8], and the hypothamic-pituitary-adrenal axis [14], inter alia. Non-photic entrainers include exercise, meals, social activity, and exogenous melatonin or serotonergic activation [16,17].

Hogenesh et al [18] suggest that phase desynchrony may be related to different phase response curves for individual tissues in response to a signal or a set of different signals. Feedback loops of intracellular transcriptional-translational gene expression regulate rhythmic protein production. Mutation in genes may result in phase lengthening, shortening, and arrythmicity. The effects of the SCN are mediated indirectly by transcription factors allowing for peripheral oscillators to oscillate with different phases. [19] Phase setting of circadian rhythms appears to be a complex activity that can be influenced by many mechanisms through numerous pathways. One method of determining synchrony of rhythms is by measuring the phase difference (PD) between two or more rhythms.

The PD is a measure of the temporal relationship between two rhythms. An optimal PD represents the temporal relationship that may result in proper functioning in the human system. A suboptimal PD is one that is greater or smaller than the optimal PD and may be associated with less than optimal functioning. A suboptimal PD may reflect a desynchrony of rhythms.

### Cortisol

Phase relationships have been studied for cortisol. Cortisol is a hormone that is expressed in the body in a circadian rhythm. In 90 per cent of healthy adults, cortisol peaks within 45 minutes of awakening, declines throughout the day and begins to rise during the night hours [20].

The peak of the cortisol rhythm may differ between healthy individuals and ill individuals. Studies of the cortisol circadian rhythm in relationship to other physiological processes have been conducted. Findings from these studies suggest that PDs exist between health and illness. Studies suggest that in Seasonal Affective Disorder (SAD), a disorder of mood, the mean cortisol rhythm itself is delayed. Avery and colleagues [21] found a phase difference between cortisol and thyroid stimulating hormone (TSH) in participants with SAD and controls. The cortisol minimum (0011 for SAD participants and 1003 for controls; *p* < 0.05) was delayed two hours in participants with SAD. While cortisol demonstrated a phase difference between the groups, the TSH phase position was not different between SAD participants and controls. Individual PDs were not reported but a two-hour cortisol phase delay in SAD participants might suggest that the cortisol-TSH PD between depressed and control participants differs.

The relationship changes between cortisol and prolactin have also been demonstrated. Koenigsberg and colleagues [22] compared 22 participants with major depression and 20 healthy controls. In addition to cortisol, the authors analyzed growth hormone and prolactin rhythms but failed to compare the individual PD between rhythms. A phase advance in the acrophase of cortisol rhythm of one hour (*p* = 0.00002) was found. There were no differences in acrophase in prolactin or growth hormone, allowing for the possibility of a phase angle difference between cortisol and prolactin and growth hormone. Results were significant despite a diagnostically heterogeneous participant group that included 69 per cent of the sample with endogenous depression, 25 per cent with psychotic depression, 38 per cent with agitated depression and 25 per cent with retarded depression.

In other studies, the changes in the phase of the sample mean of cortisol does not vary in illness states. In these studies, other rhythms such as immune factors, growth hormone and prolactin are advanced or delayed relative to mood. Authors of these studies fail to analyze the individual rhythm relationships, but mean differences may suggest the possibility of individual PDs in health and illness. Alesci and colleagues [23] studied the relationship between cortisol and plasma IL-6 levels. The mean phase position of both cortisol and IL-6 in depressed and non-depressed participants were reported. PD differences were found in IL-6 but not cortisol.

Studies have been conducted to explore the timing of endogenous rhythms and sleep quality parameters, such as sleep onset. In an early study, the PD between the cortisol and the sleep rhythm was reported [24]. Depressed participants demonstrated a smaller PD (*p* = 0.017) between cortisol nadir and sleep onset (188 minutes) compared with controls (239 minutes).

### Estradiol

Estradiol demonstrates a circadian rhythm. The diurnal cycle of estradiol exhibits an early morning peak and two, three or four ultradian harmonics throughout the 24-hour period [25]. During the menstrual phase, the peak in estradiol occurs later in the morning. The normal character of the estradiol rhythm is relatively unaffected by the menstrual cycle, except for the acrophase during the menstrual phase.

Studies involving the circadian rhythm of estradiol alone are few. Two studies have compared estradiol and cortisol circadian rhythms. Taleb, Krause and Goretzlehner [26] investigated cortisol and estradiol rhythms in women with preterm labor. They found that the cortisol rhythm was phase delayed in preterm labor compared to term labor. The estradiol rhythm did not differ in phase position between preterm labor and term labor. The phase shift of cortisol in the absence of a similar phase shift in estradiol suggests a possible misalignment between the rhythms.

In a study by Bao and colleagues [27] circadian cortisol and estradiol rhythms in 27 women, 12 with a diagnosis of major depression were compared. As expected, both cortisol and estradiol demonstrated clear diurnal rhythms. While in the control group the acrophases of cortisol and estradiol correlated, the acrophases in the depressed group demonstrated no correlation the late luteal phase. This may suggest that a coupling of cortisol and estradiol is present in healthy participants but not depressed women. A decoupling of the cortisol and estradiol phases may suggest a phase misalignment in depressed women but not healthy controls.

Through the findings of multiple studies, evidence has accumulated regarding the changing environmental milieu of reproductive hormones. The various phases of the menstrual cycle influence the actions of circadian rhythms. Premenstrual syndrome and premenstrual dysphoric disorder are characterized by changes in positive and negative affect, occurring predominantly during the luteal phase of the menstrual cycle [28,29,30,31]. In other studies conducted of the menstrual cycle, it has been suggested that cortisol secretion varies over the cycle [32,33].

Research in PDs has been limited to the relationship between exogenous rhythms such as timing and intensity of light, temperature, humidity, sleep/wake, sound and a single endogenous rhythm [34]. Few researchers have investigated the PDs among multiple endogenous rhythms. Specifically, limited studies have been conducted to determine optimal PDs between cortisol and estradiol in relation to affect. Therefore, the purpose of the current study was to investigate the relationship between the synchrony of estradiol and cortisol, two hormonal rhythms, and affect in premenstrual white women.

## Methods

### Design

A descriptive, comparative, correlational study design was used to explore the phase relationships between the biological rhythms of cortisol and estradiol and the correlation of this relationship with affect.

### Sample

Twenty-four women with normal menstrual cycles were recruited for this study. Most of the participants (n = 22) were from a population of urban university students. Two additional participants were community dwelling ambulatory women who resided in urban and rural areas of southeast Michigan. Participants were recruited through invitation by the researcher at graduate and undergraduate classes at the university. It was emphasized that the participation would not in any way affect course grades. Additional recruitment from the community was needed, as adequate sample size was not obtained through university recruitment. This was accomplished through flyers posted in public locations and direct approach by the investigator. Individual participants were informed of the study design, procedures, participant responsibilities and compensation. Written consent was obtained from all participants.

**Inclusion criteria.** Eligible participants had the following characteristics: a premenopausal female between 25 and 35 years of age; regular menstrual cycles between 27–32 days; White; able to read and speak English; nonsmoker, or willing to refrain from smoking during data collection; and major sleep period that occurred during the night.

**Exclusion criteria.** Exclusion criteria included: pregnancy or lactation within the past three months; prescription drug use including oral contraceptives within the last three months; steroid use; illicit drug use; pre-existing diagnosis of any psychiatric disorder; pre-existing diagnosis of an endocrine disorder; pre-existing diagnosis of sleep apnea or periodontal disease; history of oophorectomy; transmeridian travel across three or more time zones in the past month; shift work in the past three months; and occurrence of unusually high stress events such as divorce, death in the family, loss of job.

### Setting

Data collection took place in the participant’s home or ordinary sphere of activity. The researcher initially met with the participant at a location convenient for the participant, to explain the study and obtain signed informed consent.

### Major Study Variables

The major variables of interest in this study were: salivary free cortisol circadian rhythm, salivary free estradiol circadian rhythm, and affect. Significant bio-markers of endocrine function included cortisol and estradiol.

**Salivary cortisol.** Cortisol reflects the functioning of the hypothalmic-pituitary-axis (HPA) and salivary free cortisol is equivalent to unbound cortisol in the body. Salivary cortisol was measured using Salimetrics’ expanded range, high sensitivity, salivary cortisol enzyme immunoassay kit (catalog number 1-3002/1-3012). This assay was designed to capture the lower levels of cortisol found in saliva when compared to serum. Intra-assay coefficients of variation range from 3.35per cent to 3.65 per cent. Inter-assay coefficients of variation range from 3.75 per cent to 6.41 per cent. Linearity of dilution tests yield recovery results from 80.1 per cent to 97.9 per cent. Sensitivity has been reported to be < 0.003 mg/dL [35].

**Salivary estradiol.** Salivary free estradiol is the biologically active form of estrogen in women of reproductive age. Estradiol has been shown to demonstrate both circadian and ultradian rhythms. Salivary estradiol was measured using Salimetrics’ high sensitivity salivary estradiol enzyme immunoassay kit (catalog number 1-3702/1-3712). The intra-assay precision is determined for high, middle and low samples. Coefficients of variation are 7.0 per cent, 6.3 per cent and 8.1 per cent, respectively. Inter-assay precision has been reported for high and low samples with the coefficients of variation of 6.0 per cent and 8.9 per cent, respectively [36].

**Affect.** Affect was measured on a bi-dimensional scale that includes positive and negative affect. Positive affect (PA) represents the degree to which an individual pleasurably engages with the environment, while negative affect (NA) represents subjective distress [37]. PA is the degree to which an individual feels alert and excited. NA is the degree to which individual feels sad and lethargic.

Positive and negative affect were measured as independent subscales using the Positive and Negative Affect Schedule (PANAS). The PANAS consists of 20 mood-based adjectives (10 to measure positive affect and 10 to measure negative affect) that the participant rates on a five-point Likert scale. Affect is measured by the participant’s subjective experience response to each of the adjectives. Participants are asked to rate the extent to which the adjectives apply to them, using subjective estimates of their being (a) not a bit, (b) a little, (c) moderately, (d) quite a bit, or (e) extremely descriptive of them. The PANAS is scored by summing the responses related to PA and summing the responses related to NA. Adjectives reflective of PA include “active”, “attentive”, and “excited”. NA adjectives include “hostile”, “afraid” and “irritable”. Higher scores on the positive affect and lower scores on the negative affect subscales are considered indicative of higher levels of positive affect. The PANAS has been used extensively in clinical and nonclinical populations to assess affect under varying temporal instructions ranging from “today” to “in general” [38,39]. Internal consistency has been reported as high, with Cronbach alphas of .88 for the PA scale, and .85 for the NA scale [40].

This study also measured affect using the Profile of Moods (POMS) subscales Tension-Anxiety and Depression-Dejection, and the POMS total score. The Tension-Anxiety subscale includes nine items measuring musculoskeletal tension and psychomotor agitation. The Depression-Dejection subscale comprises 15 items measuring personal inadequacy, hopelessness, sadness, isolation and guilt. A global estimate of mood is given by the summation of the six subscales where the Vigor-Activity subscale is weighted negatively. Internal consistency for all subscales has been reported at .90 and above. Test-retest reliability ranges from .65 for Vigor to .74 for depression [41].

### Data Collection Procedure

This study was approved by Wayne State University Institutional Review Board, protocol number 0911007749. Potential participants were approached by the principal investigator individually and in classroom settings at a Midwest urban university. After consent was obtained, the luteal phase of their menstrual cycle was determined from their previous menses. Collection of samples began between day 25 and 28 of the menstrual cycle. On the day of collection, each participant completed the demographic questionnaire, the PANAS, and the POMS. These were collected once on the first day of collection. As affect can vary over the day by as much as 10 points on a 100 point scale [42], with the nadir correlated with the body temperature nadir [43], the POMS and PANAS were administered in the early afternoon. Participants then received instruction in keeping a diary and the salivary sampling protocol. The diary consisted of columns with the following headings: awake time, first collection time, any food eaten 60 minutes prior, second collection time, any food eaten 60 minutes prior. The column headings repeated for a total of thirteen collection times. In addition, the diary asked the participant to record time of sleep, caffeine intake, and alcohol intake, for each collection time.

The sampling protocol for cortisol and estradiol was as follows. At every collection, the participant was asked to refrain from brushing or flossing the teeth until after the second collection of the day. Participants were not to eat within the hour before collection. Immediately prior to collection the participant rinsed her mouth with cool water. After a five-minute wait, the participant expectorated through the straw provided into the sampling container provided.

On the collection day, the participant was instructed to perform the sampling protocol at time of awakening, 30 minutes later, and then every two hours around the clock for the remainder of the day, for a total of 13 samples. Collection materials were kept at the bedside during the night and the participant was instructed to collect the sample in darkness while remaining in bed. Samples were kept on ice or refrigerated until retrieved by the researcher. Participants recorded collection times and any deviations from protocol in a diary.

**Salivary Cortisol and Estradiol Collection.** Salivary sampling employed a passive drool technique in which approximately 1.8 mL of saliva was collected by drooling down a straw into a collection vial according to manufacture recommended protocol. After retrieval from the participants, samples were frozen to 0 degrees Fahrenheit until analysis.

Assays were run in duplicate on 310 salivary samples using Salimetrics High Sensitivity Salivary Cortisol Enzyme Immunoassay Kits [35]. and Salimetrics High Sensitivity Salivary Estradiol Enzyme Immunoassay Kits [36]. Salivary cortisol and estradiol levels were determined by calculating the mean of the duplicate assay results. The quantitative measurement of cortisol and estradiol was determined by using an enzyme-linked immunoabsorbent technique (ELISA) according to manufacturer’s instructions [35,36]. The intra-assay precision for the cortisol assays was reported as 0.999 (*SD* = 0.033) μg/dL for high values and 0.097 (*SD* = 0.004) μg/dL for low values. The coefficients of variation were 3.35 and 3.65, respectively. The lower limit of sensitivity for cortisol was 0.003 μg/dL. The cortisol inter-assay precision was determined to be 1.020 (*SD* = 0.038) μg/dL for high values and 0.101 (*SD* = 0.006) μg/dL. Coefficient of variation was 3.75 and 6.41, respectively [35].

The intra-assay precision for the estradiol assay kits were reported as 20.26 (*SD* = 1.42) pg/ml for high values, 7.24 (*SD* = 0.45) pg/ml for mid-range values and 3.81 (*SD* = 0.31) pg/ml for low values. Coefficients of variation were 7.0 per cent for high, 6.3 per cent for mid and 8.1 percent for low values. Inter-assay precision was 24.62 (*SD* = 1.47) pg/ml for high values and 4.76 (*SD* = 0.42) pg/ml for low values. Coefficients of variation were 6.0 per cent for high values and 8.9 per cent for low values. The lower limit of sensitivity for estradiol is 1.0 pg/ml [36].

## Data Analysis

All statistical analyses were completed using the Statistical Package for the Social Sciences (SPSS) version 19.0 for PC [44] and GraphPad Prism 5.0 for MacOS [45]. Descriptive statistics, including mean, mode, standard deviation, range and skewness were used to describe the characteristics of the sample and study variables. SPSS [44] was used to compute the descriptive statistics.

## Results

**Figure 1:**
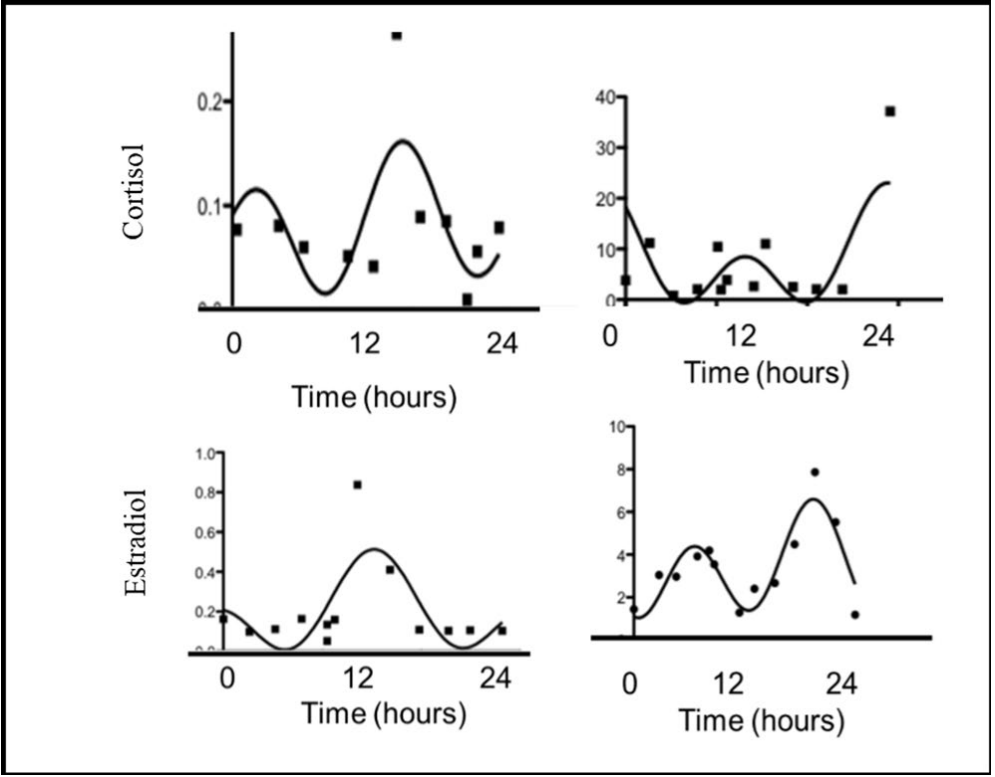
Cortisol and Estradiol Curve Fit.

Variables were examined to meet the assumptions of linear and nonlinear regression and correlation analysis. Nonlinear regression was performed using GraphPad Prism 5.0 [45]. GraphPad Prism employs the Marquardt method of performing nonlinear regression. Automatic outlier elimination was performed using a Q value of 1 per cent. Curve fitting was tested by visual examination of the raw data (see Figure 1) and curve fitting using GraphPad Prism 5.0d constrained nonlinear regression analysis. The model selected was a multiple cosinor curve Y = M + A*cos(X-phaseshift) + B*cos(C*(X-d)), where M is the mean of the circadian rhythm and A is the circadian rhythm amplitude. B is the amplitude of the ultradian rhythm and C is the harmonic, where the second harmonic is equal to eight hours, the third harmonic is six hours and the fourth harmonic is four hours. Finally, d is the phase position of the ultradian component.

Curves of two, three and four harmonics were compared using Akaike’s Informative Criterion (AIC) and the curve harmonic and the best fit was chosen. Independence of the variables of amplitude, mesor and phase was determined using descriptive and Pearson product-moment correlation statistics. The PD between cortisol and estradiol was determined by subtracting the estradiol acrophase from the cortisol acrophase. For values greater than 12 hours, 24 was subtracted from the value, and for values less than –12 hours, 24 was added to the value to account for the circular nature of clock time. The cortisol-estradiol PD was regressed against the health indicators of affect using the quadratic equation Y = B0 +B1*X +B2*X^2. The quadratic model was compared to a straight line using the Extra Sum of Squares Fit Test. A *p*-value of 0.05 or less was used to determine significance. For models that fit a quadratic equation significantly better than a straight line, the cortisol-estradiol PDs were examined for equivalency.

Initially, 24 participants were recruited for the study. One participant was subsequently removed from the analysis due to the inability to determine an estradiol acrophase. The final sample consisted of 21 (91.3 per cent) participants from the college of nursing and two (8.7 per cent) from the community. Sample characteristics are presented in Table 1. The mean age of the sample was 28.7 (*SD* = 5.8) years with an inclusive range of 21 to 39 years. The mean BMI was 24.7 (*SD* = 4.5) Kg/m^2^ with a range from 18.0 to 41.6 Kg/m^2^.

**Table 1:** Descriptive Statistics.

	Total Sample (n = 23)		
Characteristic	Mean (*SD*)	Range	Number (%)

Age	28.7(5.8)	21–39	
BMI	24.7(4.5)	18.0–41.6	
Marital Status			
Married			6(26.1)
Single			6(69.6)
Divorced/Separated			1(4.3)
No. Children^1^			
None			15(65.2)
1 to 5 years			3(13.0)
6 to 12 years			4(17.4)
Education			
High School			5(21.7)
Bachelor’s Degree			16(69.6)
Master’s Degree			2(8.7)
Daily Caffeine (cups)	1.3(1.1)	0–4	
**POMS**			

Total Scale Score	15.4(24.1)	–24– + 86.8	
Depression-Dejection Subscale Score	5.2(6.9)	0–25	
Tension-Anxiety Subscale Score	6.3(4.2)	0–16	
Fatigue-Inertia Subscale Score	7.3(5.4)	0–23	
Vigor-Activity Subscale Score	14.6 (5.5)	3–25	
**PANAS**			

Positive Affect Subscale	33.6(8.3)	13–46	
Negative Affect Subscale Score	17.3(5.7)	10–30	
SSQ Scale Score	4.5(.97)	1.71–5.93	
PSQI Scale Score	3.5(1.7)	1–8	
Energy VAS-Energy Scale Score	72.7(14.8)	45.3–113.3	
Cortisol Acrophase (hours)	9.2(3.4)	2.06–15.01	
Estradiol Acrophase (hours)	9.9(7.4)	.19–22.19	
Cortisol-Estradiol PAD (hours)	2.7(5.0)	–7.9–11.92	

^1^ n = 22 for this variable.

The mean scores on the two PANAS subscales and the POMS can be found in Table 1. Cronbach’s alpha in this study for the PANAS positive affect subscale was .92. Cronbach’s alpha for the PANAS negative affect subscale was .86. On the POMS scale, Cronbach’s alpha for the total scale was .81, with a Cronbach’s alpha of .90 for the Depression-Dejection subscale, and .79 for the Tension-Anxiety subscale.

Cortisol was measured using the Salimetrics High Sensitivity Cortisol Enzyme Immunoassay. The Salimetrics High Sensitivity Salivary Cortisol Enzyme Immunoassay has a sensitivity to detect 0.003 μg/dL, with serum correlation of 0.9. The intra-assay coefficients of variation for this study were 6.7 for cortisol and the inter-assay coefficients of variation were 11.9. Estradiol was measured using the Salimetrics High Sensitivity 17β-Estradiol Enzyme Immunoassay. The Salimetrics High Sensitivity Salivary 17β-Estradiol Enzyme Immunoassay has a sensitivity of 0.01 pg/mL, with serum correlation of 0.80. The intra-assay coefficients of variation for this study were 9.32 and the inter-assay coefficients of variation were 13.3. For the estradiol samples, the pH indicator in the assay diluent indicated a possible saliva pH outside of acceptable parameters. A pH test was performed on a random 15 per cent of the saliva samples. None of the pH values were below the acceptable value of 5. Six (19.3 per cent) random samples were slightly higher than the acceptable upper limit of nine with values ranging from 9.03 to 9.64. Elevated pH may artificially lower the estradiol values.

The mean cortisol-estradiol PD of the full sample is reported in Table 1. The cortisol-estradiol PDs and the health measures were then modeled to the equation Y = B0 +B1*X +B2*X^2. Goodness of fit was determined using the *R*^2^ values, D’Agnostino’s normality of residuals, run tests and visual inspection of the data points (Table 2).

**Table 2:** Goodness of Fit for Cortisol and Estradiol Curve (N = 23).

	Cortisol Curve				Estradiol Curve			

Participant	*R*^2^	Normality of residuals	*p*-Value	Run Test *p*-value	*R*^2^	Normality of Residuals	*p*-value	Run Test *p*-Value

1	0.47	18.14	0.000*	0.296	0.46	0.042	0.980	0.966
2	0.55	.794	0.672	0.296	0.40	1.333	0.513	0.999
3	0.95	1.067	0.587	0.976	0.72	0.105	0.949	0.881
4	0.32	17.75	0.000*	0.576	0.68	0.340	0.843	0.043*
5	0.46	2.973	0.226	0.911	0.51	1.359	0.507	0.733
6	0.52	7.302	0.026*	0.576	0.55	0.349	0.839	0.966
7	0.88	1.612	0.447	0.533	0.56	1.987	0.371	0.954
9	0.77	7.756	0.021	0.606	0.74	0.889	0.641	0.966
10	0.63	0.229	0.892	0.347	0.54	6.045	0.049*	0.929
11	0.73	1.940	0.379	0.879	0.57	5.175	0.075	0.347
12	0.56	1.182	0.554	0.999	0.66	1.687	0.430	0.966
13	0.75	3.372	0.185	0.879	0.45	3.575	0.137	0.879
14	0.78	1.431	0.489	0.347	0.35	2.246	0.325	0.966
15	0.54	4.246	0.120	0.878	0.67	1.577	0.454	0.793
16	0.73	0.601	0.740	0.576	0.49	1.444	0.486	0.347
17	0.55	1.182	0.554	0.879	0.38	0.858	0.651	0.966
18	0.62	0.015	0.993	0.500	0.50	0.480	0.787	0.733
19	0.75	1.438	0.487	0.500	0.37	1.287	0.526	0.966
20	0.57	3.876	0.144	0.879	0.49	3.822	0.148	0.999
21	0.90	0.999	0.607	0.879	0.52	0.356	0.837	0.500
22	0.60	11.150	0.004*	0.652	0.88	5.200	0.074	0.47
23	0.69	11.850	0.003*	0.348	0.29	0.258	0.879	0.793
24	0.83	2.186	0.335	0.296	0.39	0.480	0.043*	0.296

*One-tailed significance level *p* < 0.05.

**Figure 2:**
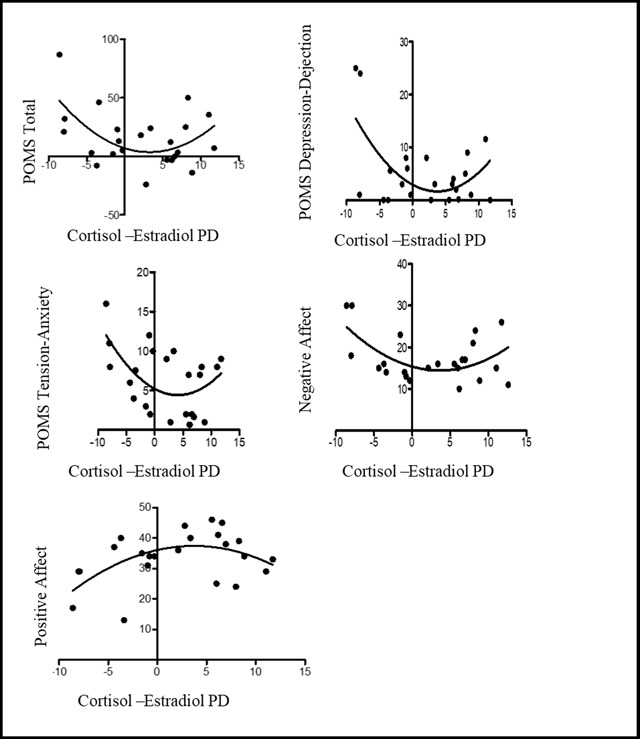
Curve Fit to Y = B0 + B1*X +B2*X^2 for Affect Measures and Cortisol-Estradiol PD.

**Affect.** The curves generated from the cortisol-estradiol PD and affect measures demonstrated data points that visually appear close to the curve in all scales (see Figure 2). Goodness of fit results can be seen in Table 3. For the sample, correlations of the cortisol-estradiol PD with the affect scales ranged from 0.28 for Positive Affect to 0.36 for the Depression-Dejection subscales. All the affect scales fit the quadratic model significantly better than a straight line at *p* < 0.05. One subscale violated the normality of residuals assumption suggesting possible systematic error. Significance was found for D’Agnostino’s normality of residuals test for Positive Affect (*K*^2^ = 7.3, *p* = 0.02). All run tests were nonsignificant in the affect measures (Table 4).

**Table 3:** Goodness of Fit for Cortisol-Estradiol PD and Affect Measures for Sample (N = 23).

	Goodness of Fit			Straight Line	
	
	*R*^2^(DF)	Normality of Residuals (*p*)	Run Test *p*	F(DFn, DFd)	*p*

Affect					
POMS Total Score	.34(20)^1^	1.4(0.50)	0.97	5.8(1,20)	0.02^2^
POMS Depression-Dejection Score	.36(20)	0.81(0.66)	0.96	6.5(1,20)	0.02^2^
POMS Tension-Anxiety Score	.30(20)^1^	5.9(0.05)	0.90	4.4(1,20)	0.048^2^
Positive Affect Score	.28(20)^1^	7.3(0.02)^2^	0.54	4.5(1,20)	0.047^2^
Negative Affect Score	.30(20)^1^	1.6(0.44)	0.51	6.21(1,20)	0.02^2^

Notes: Model: Y = B0 + B1*X + B2*X^2. POMS = Profile of Moods; ^1^ Correlation greater than 0.25; ^2^ Significant at *p* < .05. ^3^ Trend to significance at *p* between 0.05 and 1.00.

**Table 4:** Optimal Cortisol-Estradiol PD Based on Curve Fit (N = 23).

Affect	

POMS Total Score	3.23
POMS Depression-Dejection Score	3.78
POMS Tension-Anxiety Score	3.90
Positive Affect Score	3.50
Negative Affect Score	3.57

PD = Phase Difference; POMS = Profile of Moods State; PD in hours

## Discussion

This study employed two questionnaires and two biological measures. Two subscales of the POMS questionnaire, and two subscales of the PANAS were the subjective measures used for this study.

### Affect

The two subscales of the Positive and Negative Affect Scales (PANAS) were used to measure affect in this study. Mean scores for this sample were 33.6 for PA and 17.3 for NA. Studies with university students found similar scores that ranged from 29 to 36 for PA and 15 to 22 for NA [40]. The Salimetrics High Sensitivity Salivary Cortisol Enzyme Immunoassay had acceptable sensitivity (0.003). The intra-assay and the inter-assay coefficients of variation were reliable at 6.7 and 11.9, respectively. Intra-assay coefficients of variation less than 10 and interassay coefficients of variation less than 15 are considered acceptable [35]. Cortisol intra and inter assay coefficient of variation were similar in other studies [46,47].

The Salimetrics High Sensitivity Salivary Estradiol Enzyme Immunoassay intra-assay and inter-assay coefficients of variation were reliable at 9.3 and 13.3, respectively. These coefficients of variation are similar to those found in other published studies [27,48].

In all participants, the cortisol and estradiol data converged on a cosinor model. Cortisol data demonstrated greater curve fit with less sum of squares differences (R-values) than estradiol. Correlation coefficients for cortisol ranged from 0.32 to 0.95 with only two data sets correlating at less than 0.50. Estradiol correlation coefficients ranged from 0.29 to 0.88 with ten data sets correlating at less than 0.50. The multioscillator cosinor model has been used to model circadian rhythm in several studies [22,25]. The cortisol data fit the curve model better than the estradiol data suggesting the possibility that the circadian and ultradian profile of estradiol expression may follow a different model from that of cortisol.

Five out of 23 data sets violated normality of residuals assumptions in the cortisol curve fit and two violated the normality of residuals assumptions in the estradiol curve fit at significance levels less than 0.05. In four of the five cortisol curves and one of the two estradiol curves the lack of normal distribution may be accounted for by an outlier. In each cortisol case the outlier may indicate the morning cortisol awakening response. In the estradiol curve, the outlier is the highest value and may represent the acrophase or may be due to measurement error. Violation of the normality assumptions suggests a systematic explanation for deviation from the chosen model. In the cortisol curves, the model may not adequately capture the cortisol awakening response. Cortisol has consistently demonstrated a robust circadian and ultradian rhythm [49,50]. Few studies have examined the circadian rhythm of salivary estradiol. Bao and colleagues [25] sampled 15 women every two hours for 24 hours at four times during the menstrual cycle, fitting the estradiol data to a cosinor rhythm. The authors found the data to fit a peaked diurnal rhythm with ultradian harmonics that demonstrated a mean acrophase in the early morning. In this current study, most participants’ estradiol curves fit the model without violating assumptions, and the correlations were lower than for cortisol. Findings of this study are consistent with Bao and coworkers [25].

The optimal PD mean value was 3.60 (*SD* = 0.26) hours, determined by the mean of the optimal PD of the five affect measures. All optimal PDs for the affect measures were between 3.23 and 3.90 hours. These findings support the those found in the literature. Several studies identified optimal PDs between hormones and the sleep parameters of wake, midsleep and dim-light melatonin onset (DLMO) in depression. Depression severity has shown a linear association with the DLMO-wake time and the DLMO-sleep time PDs in 18 depressed women. Depression severity demonstrated a linear relationship between temperature-midsleep PD and the DLMO-temperature PD, however no group differences were noted [51]. PD between temperature minimum and wake time in 43 SAD participants suggested a trend toward a three-hour PD associated with reduction in symptoms after light treatment that was not statistically significant [52]. Group differences in cortisol-DLMO were found in six healthy and six depressed individuals, with approximately a two-hour greater PD in depressed participants [53]. In addition, a six-hour optimal PD was demonstrated between DLMO and mid-sleep in winter depression [54]. Except for Buckley and Schatzberg [53], no studies have investigated an optimal PD between two endogenous hormones.

### Strength

This study was the first known to investigate the PD between cortisol and estradiol and the relationship between the cortisol-estradiol PD and affect. This study has several strengths. In this, cortisol and estradiol were measured every two hours across an entire 24-hour period. The cortisol awakening response was captured by an additional saliva collection 30 minutes following wake time for a total of 13 saliva samples per participant. Multiple sampling across the 24-hour period allows for greater confidence in modeling the circadian and ultradian rhythms. Another strength was the use of multiple measures for affect. To reduce confounding variables the sample was homogenous for race and occupation. Saliva samples were obtained at the same time in the luteal phase of the menstrual cycle for all participants, avoiding differences in circadian rhythm characteristics due to phase of the menstrual cycle. This study was further strengthened by the natural setting in which saliva was collected. A natural setting allows for hormone expression in the body that is more consistent with the participant’s daily secretion patterns. This study provided insight into the relationships between circadian rhythms within the individual, as opposed to aggregate means.

### Limitations

This study was limited by several factors. First, the relationships tested must be understood as associations, not causal relationships. Generalizability is limited by the homogeneity and small size of the sample. The convenience sample was selected primarily from a cohort of graduate and undergraduate nursing students at an urban university. Education level, student status and race were similar across the sample. The sample consisted of 23 women; too small for adequate power to determine group differences. Non-significant findings may be a result of type II error and significant findings may possibly be spurious due to the small sample size.

Another design limitation was the sampling method of every two hours over a single 24-hour period. It is optimal to sample salivary hormone over several days and take the mean values to more accurately model the circadian rhythms. Numerous studies that employ salivary samples across the day have been limited to two to six samples. Study designs that use laboratory conditions and plasma sampling provide the opportunity to perform sampling at greater frequencies. The optimal number of salivary samples needed for both adequate curve fit and minimizing interruption to normal daily activities has not been adequately studied. Sampling every two hours has been suggested to be acceptable, however, a higher sampling rate may provide greater confidence in the rhythm parameters.

Subjective measures of affect, sleep quality and energy level, and self-report compliance threaten validity. Subjective measures have been found to inconsistently correlate with objective measures [53]. Sampling in a natural environment prohibits researcher oversight of participant compliance with study procedures. Abnormally high pH values in 19 per cent of the estradiol samples may represent an additional threat to assay validity from potentially contaminated specimens.

## Conclusions

In this study the circadian characteristics of two hormones in healthy women and their relationship to affect were investigated. This study endeavored to explain the mechanisms by which affective disorders emerge from the interplay of various circadian rhythms. Understanding the PDs among rhythms in humans holds the potential to understand the development of the symptoms that are common to many disease processes. This study contributes knowledge by suggesting a possible phase relationship between cortisol and estradiol that is implicit in lower affect in healthy women. Lower affect may be a significant contributory symptom in the development of depression and other affective disorders.

Based on the possible relationship between the cortisol-estradiol PD and affect, phase shifting interventions can be developed and tested to determine their effects on depression, premenstrual syndrome, and premenstrual dysphoric disorder, inter alia. The emerging model may suggest phase responses between cortisol and estradiol may differ based on the specific entrainer. The possibility that cortisol may represent an arousal-dependent rhythm while estradiol may represent an arousal-independent rhythm is suggested. Arousal-independent phase shifters include melatonin and g-*Aminobutyric acid* (GABA). Arousal-dependent non-photic phase shifters include serotonin [55]. Explicating a model for phase-setting in human health provides a method by which to explore additional, yet unknown, phase entrainers. Measuring the effects of entrainers on an optimal cortisol-estradiol PD can contribute to understanding the potential role of interventions in the alleviation of symptoms of illness. Potential entrainers may include such diverse phenomena as music, visual art, and physical/temporal order or disorder among many others. Much more research is needed to understand the effects of specific entrainers on affective disorders.
